# Stakeholder perspectives on large-scale marine protected areas

**DOI:** 10.1371/journal.pone.0238574

**Published:** 2020-09-02

**Authors:** Evan Artis, Noella J. Gray, Lisa M. Campbell, Rebecca L. Gruby, Leslie Acton, Sarah Bess Zigler, Lillian Mitchell

**Affiliations:** 1 Department of Geography, Environment and Geomatics, University of Guelph, Guelph, Ontario, Canada; 2 Nicholas School of the Environment, Duke University, Beaufort, North Carolina, United States of America; 3 Department of Human Dimensions of Natural Resources, Colorado State University, Fort Collins, Colorado, United States of America; University of Waikato, NEW ZEALAND

## Abstract

Large-scale marine protected areas (LSMPAs), MPAs greater than 100,000km^2^, have proliferated in the past decade. However, the value of LSMPAs as conservation tools is debated, in both global scientific and policy venues as well as in particular sites. To add nuance and more diverse voices to this debate, this research examines the perspectives of stakeholders directly engaged with LSMPAs. We conducted a Q Method study with forty LSMPA stakeholders at five sites, including three established LSMPAs (the Marianas Trench Marine National Monument, United States; the Phoenix Islands Protected Area, Kiribati; the National Marine Sanctuary, Palau) and two sites where LSMPAs had been proposed at the time of research (Bermuda and Rapa Nui (Easter Island), Chile). The analysis reveals five distinct viewpoints of LSMPAs. These include three more optimistic views of LSMPAs we have named *Enthusiast*, *Purist*, and *Relativist*. It also depicts two more cautious views of LSMPAs, which we have named *Critic* and *Skeptic*. The findings demonstrate the multi-dimensionality of stakeholder viewpoints on LSMPAs. These shared viewpoints have implications for the global LSMPA debate and LSMPA decision-makers, including highlighting the need to focus on LSMPA consultation processes. Better understanding of these viewpoints, including stakeholder beliefs, perspectives, values and concerns, may help to facilitate more nuanced dialogue amongst LSMPA stakeholders and, in turn, promote better governance of LSMPAs.

## Introduction

In the past decade, the ‘race’ to establish large-scale marine protected areas has been a dominant trend in marine conservation practice [1]. Large-scale marine protected areas (LSMPAs) are defined as any area greater than 100,000 km^2^ that is managed for conservation purposes. More than twenty LSMPAs have been established since 2006, constituting more than 70% of the global coverage of MPAs [2]. The four largest MPAs have been created or expanded since 2016 [3]. Some scholars have expressed support for LSMPAs, noting their important role in a comprehensive plan for ocean conservation [4–6], while others have expressed concern over the primacy, efficacy and equitability of this approach [1, 7]. For some, LSMPAs are key to meeting global conservation targets, such as the Convention on Biological Diversity Aichi Target 11, which calls for 10% of ocean space to be protected by 2020 [8]. However, others are concerned that this drive to meet targets has resulted in a race for quantity of MPAs over quality, or in the case of LSMPAs, size over substance [9]. There is an ongoing, polarized, and high-profile global debate over the rationales for and impacts of LSMPAs, in the scientific community as well as among conservation practitioners [10], in popular media outlets [7, 11] and in international policy venues [12]. Contributors to the global debate include scientists (both biophysical scientists and social scientists), representatives of non-governmental organizations (NGOs), and politicians.

In the scientific literature, arguments for and against LSMPAs relate to their ecological, economic, political, sociocultural, and management/design impacts and features. In some cases these arguments depend on the level of protection afforded by a LSMPA, while in other cases they apply to all LSMPAs. Scientists have identified multiple ecological benefits and advantages of LSMPAs, particularly no-take LSMPAs, including the protection of entire ecosystems, especially those not normally protected by smaller MPAs [4, 5, 13], conservation of migratory species not normally protected by smaller MPAs [14, 15], ability to withstand large-scale disturbances such as climate change [4–6], protection of marine wilderness [16] and provision of ecosystem services, including fisheries [6]. In contrast, others suggest LSMPAs do not protect the most threatened ecosystems or species [17–19], and may even result in environmental harm outside of their boundaries, by shifting fishing effort elsewhere [20]. LSMPAs may also disproportionately contribute to achieving area-based targets for marine protections (e.g., 10% in Aichi Target 11), while adding little to the protection of a representative, well-connected system of MPAs [1]. It is also noted that the ecological outcomes of LSMPAs are not yet known and are difficult to monitor [21, 22].

The economic costs of LSMPAs, both absolute and relative to other efforts, are also debated, in addition to the economic impacts and rationales of these sites. Some scientists argue that LSMPAs are more cost effective compared to smaller MPAs, both in terms of establishment and management costs [6, 23, 24], and that LSMPAs can be used to promote tourism and alternative socio-economic benefits as well as attract conservation donors [5]. And while LSMPAs can be expensive to manage, monitor and enforce [5, 25], this is also true of smaller MPAs [6]. However, others argue that LSMPAs divert limited resources away from the most threatened areas (typically inshore/coastal areas) to remote, offshore areas that are least in need of protection [1, 19, 20, 25], although there is a lack of evidence that LSMPAs are in fact diverting resources away from smaller, coastal MPAs [6, 26].

Some scientists have been particularly concerned with the politics of LSMPAs, suggesting states establish LSMPAs primarily for political reasons such as meeting international targets [1, 21], building environmental legacies for political leaders [1], or asserting sovereignty and advancing geopolitical agendas [27–29], rather than to achieve conservation outcomes. There is concern that LSMPAs can alienate stakeholders and increase geopolitical inequities [9, 21, 30]. In contrast, other scientists have argued that governments should be commended rather than critiqued for their marine conservation efforts [26], with many noting the important role that LSMPAs play in meeting global targets, and therefore advancing the overall goal of marine conservation [22, 31].

There are also debates regarding the sociocultural impacts of LSMPAs. Some scientists suggest LSMPAs can perpetuate colonial conservation practices and restrict local and Indigenous rights [30, 32], noting that even remote LSMPAs can undermine social justice [9]. However, others suggest that LSMPAs may protect sites with unique cultural and historical heritage [5, 13, 33] and ensure the recognition of Indigenous knowledge, values and rights to resource use [6, 33]. Recognizing the importance of social justice, they assert that LSMPAs can address justice and equity concerns when appropriately managed [6]. It is also suggested that because LSMPAs generally affect fewer stakeholders than smaller, coastal MPAs, they are less likely to lead to social injustices [26]. It is clear that LSMPAs can have a range of social outcomes, both positive and negative, even when they are remote [34].

Finally, there are debates regarding the design and management of LSMPAs. Some suggest that a single no-take LSMPA is better for meeting conservation goals and easier for monitoring and enforcement than networks of smaller MPAs or zoned LSMPAs [15, 35], while others are concerned about the top-down process in which most LSMPAs have been established [9, 30]. Monitoring and enforcement are also a point of debate, with some suggesting these can be achieved in LSMPAs via collaborative partnerships between sites and countries using advanced satellite monitoring technologies [13, 36], and others highlighting the increased challenges of surveillance at this scale [37].

One issue that most scientists agree on is that more research is needed to inform these debates [4, 12, 38]. It is unclear, for example, whether and how stakeholders experiencing LSMPAs in particular places (including all phases of LSMPA negotiation, declaration, establishment, and implementation) align with these arguments for and against LSMPAs identified in the global debates. Stakeholders associated with smaller, inshore MPAs hold a range of viewpoints, which can lead to conflict, disagreement, and poor outcomes [39–41]. However, several scientists suggest that LSMPAs have fewer stakeholders and interests than smaller MPAs, implying that conflict is less likely and human dimensions less important to consider [24, 26]. In contrast, conservation social scientists have emphasized the importance of considering the human dimensions of conservation generally [42] and LSMPAs specifically [12, 43, 44], including documenting people’s perceptions of conservation initiatives as evidence to inform decision-making processes [45, 46]. In this paper, we examine stakeholder perceptions of LSMPAs as policy tools through a comparative study across five LSMPA sites, in order to achieve two goals. First, we consider how global debates about the purpose and utility of LSMPAs are reflected in stakeholders’ perspectives. Ultimately, it is stakeholders’ perspectives that influence whether conservation policies are supported in particular places. When deciding how to manage existing LSMPAs or whether to establish new ones, it is essential that stakeholders’ perspectives are also considered. Second, we seek to expand the global LSMPA debate, by including a wider range of voices and viewpoints. Stakeholders’ perspectives offer important evidence regarding the validity and influence of claims about LSMPAs.

## Methods

### Q methodology

Q methodology is a mixed quantitative and qualitative research technique designed to elicit people’s viewpoints on a particular topic [47]. It uses factor analysis to statistically identify patterns within participants’ rank-sorting of items. These patterns constitute distinct viewpoints and can be interpreted and compared to one another [48]. Q methodology has been increasingly used for conservation research in recent years (e.g. Holmes et al. 2017, Gall and Rodwell 2016), including for the assessment of management alternatives, critical reflection, policy appraisal, and conflict resolution [49]. While it is often used to understand viewpoints in one case study site, it can also be used to analyze viewpoints across multiple locations and sites [50, 51].

We used Q methodology to examine viewpoints of diverse LSMPA stakeholders in five sites: Bermuda, where a consultation process took place from 2010 to 2015 on a proposed LSMPA, but an LSMPA has not been established; Rapa Nui (Easter Island, Chile), where an LSMPA was announced in 2015, but not approved until 2017 (after this research was completed, and following an Indigenous consultation process); a recently declared LSMPA in Palau (the Palau National Marine Sanctuary, est. 2015); and two longer established LSMPAs, in Kiribati (the Phoenix Islands Protected Area, est. 2008) and in the Commonwealth of the Northern Mariana Islands (CNMI) and Guam, U.S.A (the Marianas Trench Marine National Monument, est. 2009). While each site has its own distinct context, design, and rules (Table 1), including variation within and among sites in level of protection, they also share several features: all were established or proposed in the past 20 years; all are (or would be) greater than 100,000km^2^; all are associated with small islands with historical or current colonial experiences, and; international NGOs were (and often still are) involved in all five sites. Q methodology allows us to explore similarities and differences in views of LSMPAs among stakeholders within and across sites, and within and among different stakeholder groups. For simplicity, we use the term stakeholder throughout, but acknowledge that some sites include Indigenous peoples who are rights-holders rather than stakeholders [43].

**Table 1:** Description of study sites.

LSMPA / Study Site	LSMPA Status at time of Research	Size (km^2^)	Site Information and Key Rules	Data Collection Sites (Year)
Bermuda	Not designated	N/A	A LSMPA was discussed between 2010–2015 but has not been designated. The most widely discussed LSMPA proposal was a no-take ring that would extend from 50 miles off Bermuda’s coast to the 200-mile Exclusive Economic Zone (EEZ) limit.	Bermuda and U.K. (2015)
Marianas Trench Marine National Monument (MTMNM)	Designated in 2009	248 517	The MTMNM includes three ‘units’: the Islands Unit protects submerged lands and waters around three remote Mariana Islands, prohibiting commercial fishing. The Trench Unit and Volcanic Unit protect submerged lands (i.e., seafloor features surrounding the deepwater Marianas Trench and certain underwater volcanic sites). Fishing is not restricted in the Trench and Volcanic Units.	CNMI and Guam, U.S.A. (2015)
Palau National Marine Sanctuary (PNMS)	Designated in 2015	500 000	The PNMS is made up of two main parts; 80% is a designated no-take reserve and 20% is a ‘domestic fishing zone’ (DFZ) that allows for the domestic sale of fish caught in the DFZ. Fishing in the no-take area will be incrementally reduced to full restriction by 2020.	Palau (2016)
Phoenix Islands Protected Area (PIPA)	Designated in 2008	408 250	The PIPA was first announced in 2006, then designated in 2008. In 2015, it was made 99.4% no-take, with a small area allowed for subsistence fishing for government staff and their families who are stationed on the island of Kanton (the only inhabited island in the protected area).	Kiribati (2016)
Rapa Nui (Easter Island, Chile)	Announced in 2015 but not yet designated	720 017	A proposal for an LSMPA was first announced by the Chilean government in 2015 pending the approval of the indigenous Rapanui people. Negotiations were undertaken from 2015–2017. Following an indigenous consultation (and after this research was completed), a multi-use LSMPA was approved in 2017, and designated in 2018.	Rapa Nui (Easter Island), Chile (2016)

### Q statements

Q methodology requires the creation of a concourse of statements that reflect the discourse about the topic of interest [48]. We identified potential statements by reviewing the peer-reviewed literature on LSMPAs, news coverage and NGO materials related to LSMPAs (such as web sites and reports), and notes and interview transcripts from research conducted at the 2014 World Parks Congress [12]. We inductively coded this material using NVivo software and categorized statements into five themes: the ecological, economic, political, sociocultural, and management/design strengths and weaknesses of LSMPAs. To reach an ideal concourse size of 40–60 statements [48], we selected 47 statements as representative of the diversity of views on these themes, editing them for clarity and to make statements relevant across sites while retaining the meaning of the original quotation (Table 4). Statements were translated into Spanish for use in Rapa Nui. We tested the Q-survey with three respondents to ensure the statements were clear and comprehensive and made minor adjustments for clarity in this pilot phase.

### Conducting Q sorts

**Fig 1:**
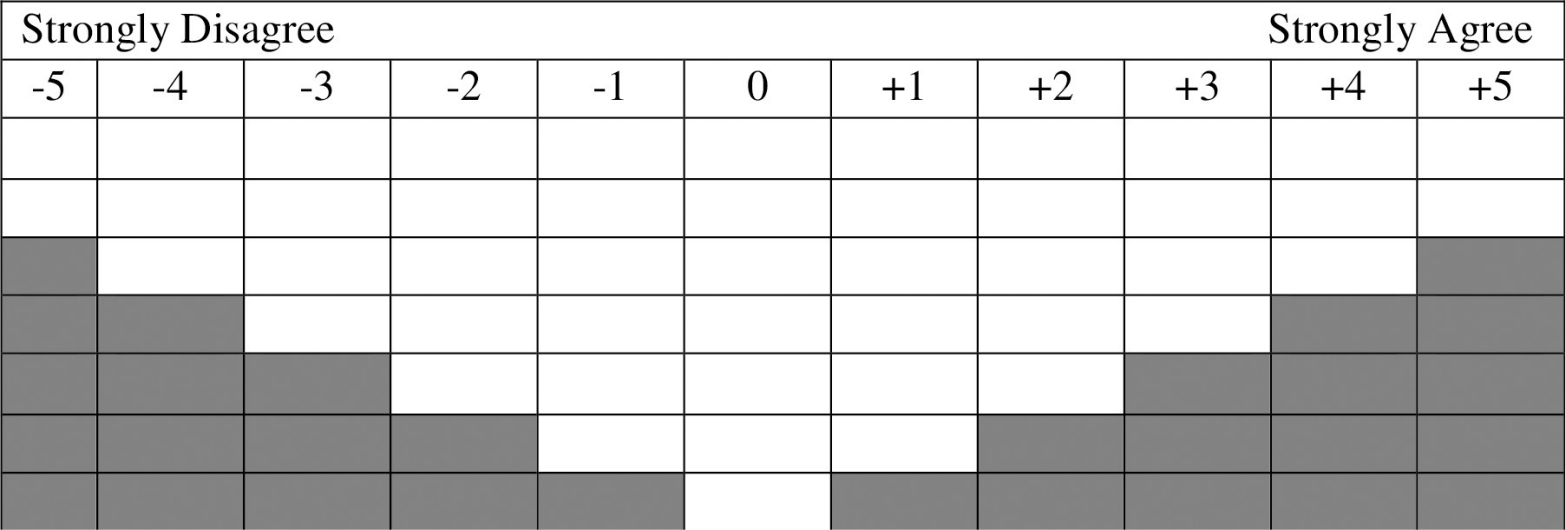
Q-sort distribution board. Participants can place two statements in the most agree (+5) and most disagree (-5) columns, while seven statements can be placed in the neutral (0) column. There is no difference in value based on the vertical order of statements in each column.

We conducted Q sorts and interviews between June 2015 and August 2016. Using purposive sampling, we identified 40 participants total (7–9 participants per site) from various stakeholder groups that had well-formed opinions on the established or proposed LSMPAs (Table 2). Our respondents included: elected representatives and employed staff of national governments as well as site-based governments (local governments or territorial governments in overseas territories or commonwealth sites); employees of both global and local NGOs or civil society groups (including Indigenous organizations at relevant sites); researchers and contracted consultants; and informed community members from industry groups such as tourism and small-scale fishing. No stakeholders from the industrial tuna fishing fleets that fish or previously fished within the waters of the PNMS (Palau) or PIPA (Kiribati) were interviewed, as members of this stakeholder group were not accessible during our field work in those countries. Participants were asked to sort the 47 statements onto an eleven-point distribution board (Fig 1) according to what extent they agreed or disagreed with each individual statement relative to other statements. We then discussed participants’ sorts during post-sort interviews. Participants were asked for their personal views, rather than official positions of their organization, employer, or government. This research received ethical approval for research with human subjects from Colorado State University and all participants gave their informed consent to participate.

**Table 2:** Summary of research participants.

Study Site	Stakeholder groups				
	Government	NGO	Researchers and Consultants	Industry and Other	Total participants per site
Bermuda	4	3	/	2	**9**
Marianas Trench Marine National Monument (MTMNM)	4	1	2	/	**7**
Palau National Marine Sanctuary (PNMS)	3	1	2	2	**8**
Phoenix Islands Protected Area (PIPA)	4	2	2	/	**8**
Rapa Nui (Easter Island, Chile)	4	3	1	/	**8**
**Total participants per stakeholder group**	**19**	**10**	**7**	**4**	**40**

### Q analysis

We analyzed the Q sorts using PQMethod software and followed criteria for analysis outlined by Watts and Stenner [48]. We first computed intercorrelations between individual Q sorts, then used Centroid Factor-Analysis to extract five factors with eigenvalues greater than 1.0. The cumulative explanatory variance of these five factors is 51%. Finally, we rotated the five factors using the automated Varimax method. To limit the number of confounded Q sorts (those that load significantly on more than one factor) we used a minimum factor loading > 0.45. Relevant Q sorts that loaded significantly on one factor were flagged as significantly loading sorts. These flagged individual Q sorts were then aggregated using PQMethod to produce factor arrays, ideal sorts that represent a viewpoint shared by a sub-set of respondents and form the basis of interpretation [48]. In order to interpret the factor arrays, we draw on data from the post-sort interviews and use illustrative quotes from respondents who belonged to that factor.

## Results

We identified five factors that account for 32 of the 40 participants’ Q sorts and reveal five distinct viewpoints on LSMPAs (Table 3). Three of the five are more optimistic views of LSMPAs, which we named *Enthusiast*, *Purist*, and *Relativist*. Two are more cautious, which we named *Critic* and *Skeptic*. Three of the remaining eight Q sorts were confounded (loaded onto multiple factors) and five Q sorts were non-significant (did not load on any of the factors). Table 3 shows how each respondent loaded on each factor, while Table 4 shows the factor arrays, or ideal Q sorts, for each of the five factors. These results illustrate several key points. First, although we found five distinct viewpoints, these are not dependent on LSMPA location. Respondents from CNMI appear in four of the five factors, respondents from Bermuda, Kiribati and Palau appear in three of the five factors, and respondents from Rapa Nui appear in two of the five factors. Second, these five points of view are shared by sub-sets of respondents at existing and proposed/stalled sites; viewpoints do not align with the status or age of a LSMPA site or proposal. Finally, there is no clear pattern among stakeholder groups, as respondents from different stakeholder groups are distributed across the different factors. In the descriptions of the five viewpoints that follow, statement numbers and the position of that statement in an ideal sort for the factor appear in parentheses, marked with a single asterisk if it is a distinguishing statement at p < 0.05 and with a double asterisk if it is a distinguishing statement at p < 0.01.

**Table 3:** Factor loadings for each participant’s Q sort.

Q Sorts		F1	F2	F3	F4	F5
Site	Stakeholder Group					
**Factor 1: *LSMPA Enthusiast***						
Bermuda	Government–Site-based	**0.4912**	0.1265	0.3468	0.1429	0.3331
MTMNM	Researcher and Contractor	**0.6007**	-0.0101	0.0259	0.3756	0.1764
Rapa Nui	Government–Site-based	**0.6828**	-0.0434	0.2121	-0.0532	-0.1548
Rapa Nui	NGO–Site-based	**0.6461**	-0.0083	0.1823	0.0872	0.2818
Rapa Nui	NGO–Site-based	**0.6334**	-0.2506	-0.1109	0.0631	0.3887
PIPA	Government–Site-based	**0.5321**	0.0799	0.0563	0.3298	0.2030
PIPA	Government–Site-based	**0.6368**	0.0310	0.1083	0.2253	0.3236
PIPA	NGO–Site-based	**0.7634**	0.0575	-0.0228	0.3198	0.0248
PNMS	Industry–Tourism	**0.7782**	-0.0785	-0.1054	0.3005	-0.0133
PNMS	NGO–Global	**0.4546**	-0.2000	0.3410	0.4076	0.1955
PNMS	Industry–Small-scale-Fishing	**0.5032**	-0.0895	-0.1322	0.1485	0.0097
PNMS	Government–Site-based	**0.4966**	-0.0909	-0.0404	0.3268	0.3218
**Factor 2: *LSMPA Critic***						
Bermuda	Government–Bermuda	-0.0992	**0.7213**	0.1977	0.2080	-0.1994
Bermuda	Industry–Other	0.2316	**0.5600**	0.2115	0.1660	-0.0594
Bermuda	Industry–Other	0.1739	**0.5876**	0.0089	0.0146	0.2493
MTMNM	Government–Site-based	-0.0497	**0.4608**	0.1025	-0.0403	0.2573
MTMNM	Researcher and Contractor	-0.2041	**0.5944**	0.3417	0.0394	-0.0040
Rapa Nui	Government–Site-based	-0.0919	**0.4622**	-0.0066	-0.0712	-0.0563
Rapa Nui	Researcher and Contractor	-0.2344	**0.6070**	-0.1074	0.1177	0.1283
Rapa Nui	Government–Chile	0.3592	**0.5699**	-0.0722	0.3180	0.0498
**Factor 3: *LSMPA Skeptic***						
MTMNM	Government–U.S.	0.2632	0.2621	**0.4863**	0.3238	-0.0121
PIPA	Researcher and Contractor	0.2961	0.1912	**0.5691**	0.3928	0.1548
PNMS	Researcher and Contractor	-0.0391	0.1266	**0.7482**	0.0629	0.0505
**Factor 4: *LSMPA Purist***						
Bermuda	Government–Site-based	0.0994	0.2220	0.1802	**0.5799**	0.2194
Bermuda	NGO–Site-based	0.4023	0.0694	0.0667	**0.7452**	0.0393
Bermuda	NGO–Site-based	0.1861	0.2156	0.2018	**0.4984**	0.0520
MTMNM	NGO–Global	0.3054	-0.3367	0.3508	**0.5324**	0.0772
MTMNM	Government–U.S.	0.2389	0.0425	-0.0066	**0.7264**	0.2228
**Factor 5: *LSMPA Relativist***						
PIPA	Researcher and Contractor	0.3415	0.1969	0.0899	0.4353	**0.4976**
PIPA	Government–Foreign	0.1575	0.1002	-0.0024	0.2444	**0.6976**
PIPA	Government–Site-based	0.4126	-0.0608	0.0353	0.2879	**0.5134**
PNMS	Researcher and Contractor	0.3385	0.3424	0.1633	0.3505	**0.4546**
**Confounded Q Sorts**						
Bermuda	Government–Site-based	0.5667	0.4948	-0.0076	0.3582	0.1261
Bermuda	NGO–Global	0.4993	-0.0974	0.1128	0.5268	0.0800
PNMS	Government–Site-based	0.5454	-0.0477	0.1201	0.5044	0.1079
**Non-significant Q Sorts**						
MTMNM	Government–U.S.	-0.1542	0.3693	0.2907	0.3114	0.1029
Rapa Nui	NGO–Site-based	0.2408	0.2582	-0.2241	0.4187	0.0620
Rapa Nui	Government–Chile	0.1662	0.3591	0.3616	-0.1972	-0.2975
PIPA	Researcher and Contractor	-0.3613	0.2568	0.1004	-0.1629	0.2614
PNMS	Government–Site-based	-0.2562	0.3662	0.2476	-0.0589	0.3472
Eigenvalues		10.8839	4.9356	1.6815	1.5127	1.5010
Explanatory variance (%)		17	10	6	12	6
Number of defining Q sorts		12	8	3	5	4

Factor loadings range from complete agreement (1) to complete disagreement (-1). Bolded numbers for F1, F2, F3, F4, and F5 are Q sorts with factor loadings >0.45 and are significant at p < 0.01.

Bolded numbers for each of the factors indicate Q sorts with factor loadings >0.45 and are significant at p<0.01.

**Table 4:** Q statements and rankings for each factor.

Q Statements	F1	F2	F3	F4	F5
1. Large MPAs have little effect on food production because they aren’t fished much.	-2	-2	-1	-2	-3
2. Large MPAs should allow cultural practitioners to conduct traditional and spiritual activities.	2	3*	1	1	1
3. Effectively managed networks of small MPAs are just as likely to achieve conservation objectives as a large MPA.	0*	-2	-3	-3	2**
4. Most large MPAs are virtually empty of people.	-1	1	1	0	5*
5. Large MPAs are a poor way to spend limited conservation funds.	-5**	-2	-1	-2	-3
6. Large MPAs provide global benefits, like protecting important fishing grounds and fish habitat.	5	0	1	4	4
7. Large MPAs are less expensive to implement per unit area than small MPAs.	-1	-3	1	0	-3
8. The recent increase in global MPA coverage achieved via the establishment of large MPAs gives a false sense of achievement.	-3	3	3	-2	1*
9. Large MPAs may be better able than small MPAs to withstand unpredictable pressures caused by climate change.	-1	-4*	0	2	2
10. Without effective enforcement, large MPAs provide a false sense of success.	2	4	5	3	3
11. Large MPAs are best suited for pristine ecosystems where exploitation is low and governments can protect them for the long term.	1	0	-1	-1	1
12. Comprehensive zoning is more likely to succeed at achieving conservation in the long-run than large MPAs.	-1	3	0	2	-1
13. Most large MPAs provide little or no protection for the species and ecosystems that are most threatened.	-4	-1	2**	-1	-4
14. Large MPAs provide an opportunity for the awakening and claiming of local or indigenous community rights.	3**	1	-4*	0	-1
15. Large MPAs protect large-scale ecosystem processes that cannot be protected with small MPAs.	2	0*	-2*	5*	3
16. Large MPAs alienate stakeholders.	-2	0	-3	-2	0
17. All relevant stakeholders, including opposing voices, should be engaged early in large MPA planning processes.	4	5	3	5	5
18. Even in remote and pristine large MPA sites, there are still relevant stakeholders of one kind or another.	0	2	1	3	4
19. The cost of large MPAs cannot be justified when compared with providing for the immediate social and economic needs of citizens.	-1	1**	-1	-1	-2
20. Large MPAs help to conserve large, migratory species.	5**	-3**	2	2	0
21. Large MPAs are put forth by coastal states to reinforce their sovereignty over sea spaces.	1	1	2	-4**	0
22. Large MPAs attract support from non-governmental and other conservation partners and donors.	2	4	2	1	3
23. Large MPAs unfairly harm hard working fishers.	-4	-1**	-4	-3	-4
24. Countries that establish large MPAs will be able to promote tourism as an alternative way to grow the economy.	3	-2	-1	1	2
25. Large MPAs with no fishing are easier to monitor and enforce than complex fisheries regulations.	1*	0*	4	3	-3*
26. By placing large parts of the ocean “off limits” for commercial activities, large MPAs are economically too costly.	-3	-1	-4	-2	-1
27. Large MPAs will result in greater fishing effort and environmental harm elsewhere.	-3	-1	-3	-3	-2
28. Equal weight should be put to ecological and socioeconomic considerations in the design of large MPAs.	1	2	0	2	0
29. The ability to enforce large MPA rules is going to get much better through new and advanced technologies.	0	1	3	4	4
30. Large MPAs in places with no local stressors are valuable as natural laboratories for scientific research.	1	2	-2	2	-1
31. Global conservation targets are the main incentive for establishing large MPAs.	0	-1	0	1	-4**
32. Large MPAs are most useful for protecting pristine marine wilderness.	2**	0	-1	0	-1
33. Large MPAs fail to serve the basic purpose of conservation if they do not limit extractive uses.	1	-1	5**	1	0
34. Large MPAs represent the interests of political elites and international NGOs, rather than local people.	-2**	4	4	-5*	1**
35. Large MPAs are better at regenerating fish stocks than small MPAs.	0	-5**	1	4**	1
36. Local residents perceive the designation of large MPAs as a loss of their inherent right to control those ocean spaces.	-1	5**	-2	0	-2
37. Large MPAs reconnect people with their cultural legacy.	4**	-4	-3	-1	0
38. Large MPAs voluntarily throw away, for free, a country’s assets.	-5	1**	-5	-4	-5
39. Large MPAs further magnify existing global inequities based on class, ethnicity, and geopolitical position.	-3	0	-5	-3	1
40. Large MPAs in remote areas divert resources from efforts to address more seriously threatened areas.	-2	2	2	-1	-1
41. Large MPAs protect geological, chemical, and oceanographic diversity.	3	-3**	0	1	2
42. Large MPAs aggressively address wide-scale, pervasive threats.	0	-5**	-2	-1	0
43. Large MPAs provide a safeguard against the threat of deep-sea mining.	0	-3**	0	0	3**
44. Large MPAs contribute little in terms of ecosystem service protection.	-4	-2	0**	-4	-5**
45. If large MPAs are established, then international donors must provide funding to offset lost revenue from extractive uses.	3**	-4	-2	-5	-2
46. Large MPAs are a precautionary approach to protect marine biodiversity.	4	2	3	3	2
47. Governments use large MPAs as an opportunity to look good politically in the global community.	-2	3	4	0	-2

F1 = Enthusiast, F2 = Critic, F3 = Skeptic, F4 = Purist, and F5 = Relativist. Statements are listed in order of the numbers on the statement cards and scores illustrate the level of agreement with each statement from -5 ‘strongly disagree’ to +5 ‘strongly agree’. Distinguishing statements are noted for each factor with a * if significant at p < 0.05 or ** if significant at p < 0.01.

### Factor 1—*LSMPA Enthusiast*

The *LSMPA Enthusiast* views LSMPAs as a triple win for conservation, delivering ecological, economic and sociocultural benefits. This view was held by the largest number of respondents, present at all sites, and held by respondents from different stakeholder groups (Table 3). Regarding ecological benefits, this viewpoint is distinct from other factors in its strong belief that LSMPAs help to conserve large, migratory species (S20**, +5) and protect pristine marine wilderness (S32**, + 2). With respect to economic issues, the *LSMPA Enthusiast* is distinguished by a strong assertion that international donors must pay to offset any losses from establishing LSMPAs (S45**, +3) and strongly disagrees that LSMPAs are a poor way to spend limited conservation funds (S5**, -5). In addition, the *Enthusiast’s* view is distinguished by a belief that LSMPAs reconnect people with their cultural legacy (S37**, +4) and provide an opportunity for the awakening and claiming of local and Indigenous community rights (S14**, +3). For example, one respondent commented how in Palau the PNMS has re-enlivened a traditional form of conservation called ‘bul’, wherein chiefs declare no fishing in a certain area. *LSMPA Enthusiasts* disagree that local residents perceive the designation of LSMPAs as a loss of their inherent right to control those ocean spaces (S36, -1). One respondent from Bermuda stated, “I think that’s nonsense […] I think they [LSMPAs] protect local rights far more”. Relatedly, *Enthusiasts* disagree that LSMPAs represent the interests of political and international NGOs, rather than local people (S34*, -2). *Enthusiasts* recognize that LSMPAs may be “insufficient” to tackle all oceans problems, as one respondent from Palau commented, but were overall most optimistic about LSMPAs.

### Factor 2—*LSMPA Critic*

The *LSMPA Critic* viewpoint is characterized by strong concern for the negative economic and sociocultural implications of LSMPAs and distrust of their purported ecological benefits. This is the only viewpoint to strongly agree that local residents perceive the designation of LSMPAs as a loss of their inherent right to control those ocean spaces (S36**, +5). In Rapa Nui, one respondent said: “My experience here is… [the Rapanui] want to protect, but they don’t want to do it in the form of an LSMPA because they feel it’s a loss of control”. Moreover, *LSMPA Critics* feel strongly that all relevant stakeholders, including opposing voices, should be heard early in the LSMPA planning process (S17 +5). This is a shared view across factors, but LSMPA critics are concerned that local voices are not heard in practice. As a respondent from Bermuda explained of the LSMPA consultation process there: “… stakeholders were not brought in early enough” and that “created a lot of anxiety and mistrust” and “really was a main reason why [the LSMPA] didn’t succeed”.

The *LSMPA Critic’s* view is also characterized by strong distrust of the purported ecological strengths of LSMPAs. This is the only viewpoint to disagree that LSMPAs are better at regenerating fish stocks than small MPAs (S35**, -5) or help to conserve large, migratory species (S20**, -3). This view is also distinguished by its strong disagreement that LSMPAs aggressively address wide-scale, pervasive threats (S42**, -5) and that LSMPAs may be better able than small MPAs to withstand unpredictable pressures caused by climate change (S9*, -4). Additionally, *Critics* are doubtful that LSMPAs protect geological, chemical, and oceanographic diversity (S41**, -3) or provide a safeguard against the threat of deep-sea mining (S42**, -5).

Lastly, the *LSMPA Critic’s* view is further distinguished by its perspective on the economic costs of LSMPAs. This is the only viewpoint to agree that LSMPAs voluntarily throw away, for free, a country’s assets (S38**, +1). *Critics* also question whether the cost of LSMPAs can be justified when compared with providing for the immediate social and economic needs of citizens (S19**, +1). As one respondent from Rapa Nui explained, the economic costs of LSMPAs “are not trivial” given other things states must provide for citizens.

While the *LSMPA Critic* view is overall the least supportive of LSMPAs, one respondent from Rapa Nui noted that “people are discussing” marine conservation more seriously now, as a result of LSMPA consultations, and these discussions are important for marine conservation. Although respondents with this viewpoint may support marine conservation generally, they do not support LSMPAs specifically.

### Factor 3—*LSMPA Skeptic*

The *LSMPA Skeptic* view is distinguished by skepticism regarding many of the purported ecological benefits of LSMPAs. In contrast to the *critic’s* dismissal of some ecological claims, the skeptic is concerned that without proper enforcement (S10, +5) or limits on extractive use within LSMPAs (S33**, +5), LSMPAs will not deliver ecological benefits and will instead provide a false sense of success. As a respondent in Kiribati explained:

“… many [LS]MPAs will be in places where there is no threat posed by any activity because that’s the only place that is politically viable to declare it […] in places where there is activity, if it doesn’t do something to actually impact on the activity then you have to ask what’s the point of actually doing it?”.

*LSMPA Skeptics* also perceive LSMPAs as representing the interests of political elites and international NGOs, rather than local people (S34 +4) and that governments use LSMPAs as an opportunity to look good politically in the global community (S47 +4). As one respondent explained “it’s a no brainer, they all love going up to John Kerry and telling them about how… great they look with their large MPAs”. (John Kerry was the U.S. Secretary of State from 2013 to 2017. In this role, he hosted the “Our Ocean” conference in 2016). So, although *Skeptics* see value in well enforced LSMPAs that limit extractive use of resources, their skepticsm results in ranking ecological benefits relatively neutral in comparison to other views (e.g. S15*, -2; S13**, +2; S44**, 0).

*Skeptics* do not see LSMPAs as providing an opportunity for the awakening and claiming of local or Indigenous community rights (S14*, -4), nor do they believe this should be a rationale for LSMPAs. Instead, as a respondent from CNMI stated, LSMPAs are “usually outside the boundaries of […] Indigenous communities’ sphere of […] jurisdiction” and so, as respondents from Kiribati and Palau stated, this is “not relevant” for LSMPAs. Nor do *Skeptics* see LSMPAs as economically too costly (S26, -4) and they strongly disagree that LSMPAs throw away, for free, a country’s assets (S38, -5). Instead as a respondent from CNMI stated, LSMPAs “protect a country’s assets”, or as a respondent from Kiribati stated, they represent a different way “of utilizing that asset”. This distinguishes the *Skeptic* from the *Critic*; the *Skeptic* would support LSMPAs that limit extractive uses and that are well enforced, and dismiss *Critics’* concerns with sociocultural and economic impacts.

### Factor 4—*LSMPA Purist*

The *LSMPA Purist* rejects political motivations for LSMPAs and, similar to the *LSMPA Enthusiast* and *Relativist*, is optimistic about their ecological potential. *Purists* strongly agree that LSMPAs may protect large-scale ecosystem processes that cannot be protected with small MPAs (S15*, +5) and that LSMPAs are better at regenerating fish stocks than small MPAs (S35**, +4). A respondent from Bermuda commented that while there was more “evidence on small MPAs being effective at managing fish stocks” they were hopeful LSMPAs would also be able to do this. Similarly, a respondent from CNMI stated they are confident that LSMPAs “should be better at improving fish stocks […] over a wider region”.

What distinguishes the *LSMPA Purist* is the importance of politics in their view, one that might be characterized as anti-politics. For example, in discussing whether LSMPAs further magnify existing global inequities based on class, ethnicity and geopolitical position (S39, -3), one respondent from Bermuda reacted very strongly, calling the statement “political bullshit”. *LSMPA Purists* are distinguished by their strong disagreement that LSMPAs represent the interests of political elites and international NGOs, rather than local people (S34*, -5) and that LSMPAs are created by coastal states to reinforce their sovereignty over sea spaces (S21**, -4). As a Bermuda respondent explained, LSMPAs are “not sovereignty issues”. Similarly, a respondent from CNMI stated, “that to me reeks of misinformation. Thinking that it’s a sovereignty issue as opposed to resource protection for the benefit of the whole”. However, while this view rejects the idea that LSMPAs are driven by political motivations, it is similar to all other perspectives in agreeing that all relevant stakeholders, including opposing voices, should be heard early in the LSMPA planning process (S17 +5).

### Factor 5—*LSMPA Relativist*

The final viewpoint is distinguished by support for LSMPAs, but not at the cost of other conservation measures. This view is distinguished by strong agreement that certain LSMPAs are virtually empty of people (S4*, +5), which means ecological benefits can be realised without causing negative economic and sociocultural impacts. For one respondent from Palau, this was relevant for the Palau National Marine Sanctuary (PNMS) specifically, as well as some other LSMPAs. He stated that “the only way to let fish stocks recover” is to have an LSMPA where there are no people. In Kiribati, one respondent claimed that PIPA being ‘empty of people’ was a large reason for selecting that site for a LSMPA.

“[i]n terms of PIPA […] we knew it was going to be virtually empty of people, there’s just the caretaker government, we’re […] going to have […] minimal impact on the local Kiribati communities”.

*LSMPA Relativists* are also distinguished in agreeing with some specific functions of LSMPAs not emphasized in other viewpoints, namely that LSMPAs provide a safeguard against the threat of deep-sea mining (S43**, +3) and can protect ecosystem services (they strongly disagree that LSMPAs contribute little to ecosystem service protection, S44, -5**). *Relativists* are distinguished by their relative neutrality on the political nature of LSMPAs, for example on whether LSMPAs represent the interests of political elites and international NGOs, rather than local people (S34**, +1), or that the recent increase in global MPA coverage achieved via the establishment of LSMPAs gives a false sense of achievement (S8*, +1). They do take one strong political stance, disagreeing strongly that global conservation targets are the main incentives for establishing LSMPAs (S31**, -4).

*Relativists* also believe that LSMPAs should not overshadow conservation in nearshore environments using other measures. They are distinguished by agreement that effectively managed networks of small MPAS are just as likely to achieve conservation objectives as an LSMPA (S3**, +2) and by disagreement that LSMPAs with no fishing are easier to monitor and enforce than complex fisheries regulations (S25*, -3). For example, a respondent from Kiribati stated that while it is true that PIPA is virtually empty of people, this should not be the only focus of marine conservation initiatives. Instead, in places like Kiribati, smaller, coastal “MPAs should also be established in areas where people” reside, in order to “assist them in enhancing, and promoting their […] food security [and] livelihoods”. Overall, *LSPMA Relativists* see the protection of remote, unpeopled sites as useful for some purposes, while also believing that more traditional conservation tools remain necessary.

Even in this *Relativist* view that sees LSMPAs as ‘virtually empty of people,’ it is still agreed that all relevant stakeholders, including opposing voices, should be engaged early in LSMPA planning processes (S17 +5). This viewpoint also strongly agrees that even in remote and pristine LSMPA sites, there are still relevant stakeholders (S18 +4). As one participant from Kiribati commented, “I think no MPA can be successful unless all the people are… engaged with in a consultative process, otherwise you just spend more money later on fixing things up”. However, *Relativists* also differentiate among LSMPAs. For example, when discussing global inequities one respondent from Kiribati said, “Where the PIPA results in global inequities–I’m just not sure how it relates to PIPA…”. The respondent went on to note how in other sites these issues of global inequities were more important, making LSMPAs “very context specific.”

### Consensus statements

Two statements (S1 and S22) met the statistical criteria for consensus statements, which do not distinguish among any of the factors. There was consensus that “large MPAs attract support from non-governmental and other conservation partners and donors.” However, this likely means different things to different viewpoints, with *Critics* concerned about the loss of local control and *Purists* seeing this as an apolitical means of supporting LSMPAs, for example. There was also consensus in the relative disagreement with the idea that “Large MPAs have little effect on food production because they aren't fished much.” Although all factors agreed that stakeholders should be engaged in LSMPA planning processes (S17), this was not a statistically significant consensus statement.

## Discussion

This article provides evidence of what stakeholders think about LSMPAs relative to global debates regarding LSMPAs as conservation tools. There are some consistencies between the stakeholder perspectives we identified and the global LSMPA debates. For example, the *Enthusiast* viewpoint aligns with many of the ecological, social, cultural, and economic benefits of these sites that are identified in the LSMPA literature. Similarly, the *Critic* viewpoint aligns with some scientists who are concerned that these sites undermine local and Indigenous rights to ocean space. The results also point to differences between stakeholders’ viewpoints and key elements of the global debates. For example, only one stakeholder viewpoint agreed that LSMPAs are ‘empty of people,’ and even for this *Relativist* viewpoint, wilderness areas are seen as the exception rather than the rule for LSMPAs. The *Enthusiast* viewpoint, which was most supportive of LSMPAs, was not characterized by a commitment to wilderness protection, which is one of the arguments for LSMPAs in the global debate [16, 35]. Another common argument in the global debate, that LSMPAs should be no-take [35], was only supported by one of the stakeholder viewpoints (the *LSMPA Skeptics*). However, this viewpoint is overall unconvinced of the value of LSMPAs as conservation tools, unlike scientists and advocates who argue for no-take LSMPAs. Overall, the five stakeholder viewpoints reveal nuanced perspectives that encompass various combinations of positions on the arguments from the global debates. It is clear that stakeholders cannot be classified as simply ‘for’ or ‘against’ LSMPAs and that it is necessary to consider how elements of the global debates fit together to form overall perspectives on LSMPAs.

The results also demonstrate that stakeholders’ perspectives may differ from how they are characterized in the global LSMPA debates. For example, according to Jones and De Santo [1], “[f]rom the perspective of national governments, it is clear that remote [LSMPAs] are win-win, in that they gain green credentials and contribute to each country’s progress towards the Aichi target.” While our results confirm a win-win view (*Enthusiasts*), it is not held solely by national government representatives, nor is it the only perspective held by national or territorial government representatives. The *Enthusiast* view, which is by far the most optimistic, is also the most heterogeneous, held by respondents from multiple stakeholder groups at all five sites. Furthermore, a number of government representatives also held the *Critic* view that does not view LSMPAs as easy ‘win-wins’. Our results suggest that government representatives hold a range of views, including those that are more critical of LSMPAs. Research must distinguish between individual views and the position of a government, in order to better understand whether and why individuals within organizations may support or oppose a conservation initiative.

A related finding is the relative insignificance of global conservation targets for informing the viewpoints of stakeholders directly engaged with LSMPAs. A main focus in global debates on LSMPAs is whether they are being established by governments simply to meet global conservation targets such as Aichi Target 11, and the consequences of this [1, 6, 18, 21]. None of the five stakeholder viewpoints is distinguished by a belief that governments use LSMPAs to meet global targets, relative to other concerns. In fact, the *Relativist* viewpoint was distinguished by its strong disagreement with this statement. While governments clearly move closer to meeting Aichi Target 11 through the establishment of LSMPAs, and may be motivated by international conservation targets, most stakeholder opinions of LSMPAs are not strongly informed by a concern with ‘target meeting’ as a driver for their establishment.

There are several implications of these results for both researchers and the broader community of practice interested in large-scale marine conservation. First, these results challenge the narrative that LSMPAs are easier to establish than smaller, inshore MPAs, due to their remote locations and fewer stakeholders and interests [24, 26]. Our results demonstrate that five nuanced, well-formed viewpoints on LSMPAs, ranging from supportive to critical, are present across multiple sites and stakeholder groups. While our study did not engage industry representatives at all sites, additional respondents (e.g. from the tuna industry) may have aligned with one of the identified viewpoints or possibly constituted an additional viewpoint. In either case, it is unlikely that more industry respondents would have resulted in fewer viewpoints. As with assumptions about government viewpoints, we would caution against assuming industry representatives are likely to align with a particular viewpoint.

Furthermore, all five of these stakeholder viewpoints share a commitment to engaging stakeholders in LSMPA planning, even for those sites that are perceived as remote and virtually empty of people. This aligns with some of the conclusions of recent empirical studies of LSMPAs, which argue that meaningful and ongoing stakeholder engagement is important for ensuring the equitability and effectiveness of LSMPAs [38, 43]. While LSMPA stakeholders may not be as numerous or as readily identifiable as in smaller MPAs [12], it is a mistake to assume that stakeholder consultation will be unnecessary or ‘easy.’

Second, and relatedly, consultation processes may be critical to informing stakeholder viewpoints and deserve greater attention. While insights regarding stakeholder consultation processes in LSMPAs are emerging [44, 52], researchers and practitioners should further consider what stakeholder engagement means and how it can be done effectively in this context. Scholars have warned that so-called ‘participatory’ approaches are not a panacea [53, 54] and have questioned whether the term is simply being used as a ‘means to an end’ rather than reflecting a genuine commitment to achieve meaningful participation in practice [55, 56]. Our results indicate that the *Critic* viewpoint was strongly influenced by dissatisfaction with consultation processes. In contrast, when stakeholders perceive processes as fair and just, then they align with an *Enthusiast* viewpoint that sees LSMPAs as providing “an opportunity for the awakening and claiming of local and Indigenous community rights” (S14).

Finally, our results suggest the need to explore how prevalent these views (and potentially others) are at particular sites, in order to inform effective decision making and stakeholder engagement. Q Methodology enables the identification of viewpoints held by a select group of people; it does not assess how prevalent these views are across entire populations. Future research could make use of survey methods to examine how common and representative the views uncovered by this study are at these sites and elsewhere. In one study, Kotowicz et al. [57] found that perspectives of the general public differed in significant ways from those of informed stakeholders. It is essential to understand how stakeholder and public perspectives interact with governance processes.

A central concern of participants at a ‘think tank’ on the human dimensions of LSMPAs was the violation of rights and perpetuation of social injustice through marine conservation [44], leading to a call for a ‘code of conduct’ for marine conservation [58]. Respecting stakeholders’ views and Indigenous rights and incorporating their viewpoints meaningfully into LSMPA governance is essential for effective and equitable conservation. By providing empirical evidence of broadly shared stakeholders’ perspectives, and the ways in which they both align with and run counter to positions identified in global debates about LSMPAs, we contribute to this goal.
